# Comparative limb proportions reveal differential locomotor morphofunctions of alligatoroids and crocodyloids

**DOI:** 10.1098/rsos.171774

**Published:** 2018-03-07

**Authors:** Masaya Iijima, Tai Kubo, Yoshitsugu Kobayashi

**Affiliations:** 1 Department of Natural History Sciences, Hokkaido University, N10W8 Kita-ku, Sapporo, Hokkaido, Japan; 2 The University Museum, The University of Tokyo, 7-3-1 Hongo, Bunkyo-ku, Tokyo, Japan; 3 Hokkaido University Museum, Hokkaido University, N10W8 Kita-ku, Sapporo, Hokkaido, Japan

**Keywords:** Alligatoroidea, Crocodyloidea, limb proportion, locomotion, feeding

## Abstract

Although two major clades of crocodylians (Alligatoroidea and Crocodyloidea) were split during the Cretaceous period, relatively few morphological and functional differences between them have been known. In addition, interaction of multiple morphofunctional systems that differentiated their ecology has barely been assessed. In this study, we examined the limb proportions of crocodylians to infer the differences of locomotor functions between alligatoroids and crocodyloids, and tested the correlation of locomotor and feeding morphofunctions. Our analyses revealed crocodyloids including *Gavialis* have longer stylopodia (humerus and femur) than alligatoroids, indicating that two groups may differ in locomotor functions. Fossil evidence suggested that alligatoroids have retained short stylopodia since the early stage of their evolution. Furthermore, rostral shape, an indicator of trophic function, is correlated with limb proportions, where slender-snouted piscivorous taxa have relatively long stylopodia and short overall limbs. In combination, trophic and locomotor functions might differently delimit the ecological opportunity of alligatoroids and crocodyloids in the evolution of crocodylians.

## 1. Introduction

Crocodylia is a remarkably successful group of large semi-aquatic predators, now thriving in the world's tropics and subtropics. The fossil record shows that the two living major groups, Alligatoroidea and Crocodyloidea (including *Gavialis* based on the molecular phylogeny, e.g. [1–3]) were split during the Late Cretaceous, and both have survived through several environmental crises afterwards, including the devastating K–Pg mass extinction event and the Plio-Pleistocene climatic deterioration [4,5].

These two crocodylian lineages are readily distinguished by the osmoregulatory organs found in the mouth. While all crocodyloids show keratinized buccal cavity and all crocodyloids except *Gavialis gangeticus* possess lingual salt glands, alligatoroids' buccal cavity is non-keratinized, and their tongues lack lingual salt glands [6–9]. This led Taplin *et al*. [7] to suggest the marine ancestry in crocodyloids, which enabled them to expand their distribution through transoceanic dispersals. Another well-known difference between the two groups is found in the sensory organs on the postcranial skin. The integumentary sense organs [10], referring to minute dark spots on scales, detect the water vibration, low-frequency bellowing sounds, water quality (pH) and possibly change in temperatures (e.g. [11–14]). These structures are present on the head as well as every postcranial scale of crocodyloids, but only on the head of alligatoroids.

Other distinctions of two major groups of crocodylians include their general differences in cranial features and minor differences in postcranial skeletons. Previous quantitative studies on cranial shape disparity of extant crocodylians demonstrated that crocodyloids occupy much larger skull morphospace than alligatoroids due to their long and narrow snouts, while the range of alligatoroids is extensively overlapped by generalized and blunt-snouted crocodyloids [15,16]. Furthermore, fossil evidence indicated that similar slender snout shapes were acquired multiple times in distantly related lineages of crocodylians, but the slender-snouted morphotype has never evolved within alligatoroids [17]. Meanwhile, recent studies on postcranial skeletons revealed that forelimb and cervico-thoracic vertebrae are significantly different between alligatoroids and crocodyloids, where alligatoroids exhibit broader and shorter cervico-thoracic neural spines and stouter coracoids and humeri than crocodyloids [18,19]. Additionally, an examination of limb muscle properties in extant crocodylians found that relative muscle fascicle lengths and muscle physiological cross-sectional areas are different between the two groups, possibly reflecting the difference in their terrestrial locomotor capabilities [20].

Consistent with the large genetic divergence between alligatoroids and crocodyloids [1,3], the above evidence related with anatomical and physiological differences between them suggested that the two groups are ecologically different [9]. However, anatomical differences that indicate functional differences between the two groups are still limited, and additional findings of such differences would be appreciated to further understand their ecological divergence. Moreover, an evolutionary correlation among the multiple morphofunctional systems that differentiated the two groups, such as feeding and locomotor systems, have yet to be evaluated.

In this study, appendicular morphology of extant and fossil crocodylians, exemplified by the length proportion of fore- and hindlimb elements, is examined. We first test if the locomotor morphologies of alligatoroids and crocodyloids are different from each other, and reconstruct their evolutionary history on phylogeny. We then investigate the correlation of locomotor and feeding morphologies to see if they evolved in association, and discuss an evolutionary connection of locomotor and feeding functions, which might jointly dictate the fate of the two superfamilies.

## 2. Material and methods

### 2.1. Specimens and measurements

The extant crocodylian sample includes 110 individuals from 20 species (table 1; electronic supplementary material, table S1), among which three individuals were adopted from Mook [22]. The sample represents all six known subfamilies (Alligatorinae, Caimaninae, Crocodylinae, Osteolaeminae, Gavialinae and Tomistominae) of the living crocodylians in the context of molecular tree [5] (table 1). Sexes were not recorded for most of the specimens and thus sexually dependent differences were not considered here, because sexes account for only limited portions of appendicular shape variation [23]. We included individuals from juveniles to large adults with various size classes (femur length ≥ 50 mm) for each species to maximize the sample size. Unavoidably, some species are represented by relatively large individuals (e.g. *Crocodylus porosus* and *Tomistoma schlegelii*) or small individuals (e.g. two species of *Paleosuchus*) compared with others (electronic supplementary material, figure S1). The fossil crocodylian sample includes taxa that are an outgroup of Alligatoroidea + Crocodyloidea (*Anteophthalmosuchus hooleyi*, *Susisuchus anatoceps*, *Borealosuchus wilsoni*, *Boverisuchus vorax* and *B. bolcensis*), basal alligatorines (*Allognathosuchus gracilis*, *Navajosuchus mooki* and *Wannaganosuchus brachymanus*), a derived alligatorine (*Alligator prenasalis*), caimanines (*Stangerochampsa mccabei*, *Necrosuchus ionensis* and *Tsoabichi greenriverensis*), a basal crocodyloid (*Asiatosuchus*) and a tomistomine (*Toyotamaphimeia machikanensis*) (electronic supplementary material, table S3).

**Table 1.** Number of extant samples examined for each skeletal element, and average rostral proportion for each species.

subfamily	species	*n* (fore- and hindlimbs)	*n* (trunk)	*n* (rostrum)	rostral proportion
Alligatorinae	*Alligator mississippiensis*	15	10	18	0.67
	*Alligator sinensis*	8	4	3	0.79
Caimaninae	*Caiman crocodilus*	9	3	10	^a^0.67
	*Caiman yacare*	5	1	9	0.71
	*Caiman latirostris*	2	1	5	0.89
	*Melanosuchus niger*	5	3	21	0.69
	*Paleosuchus palpebrosus*	5	3	5	0.62
	*Paleosuchus trigonatus*	2	n.a.	2	0.57
Crocodylinae	*Crocodylus acutus*	7	4	25	0.47
	*Crocodylus johnsoni*	7	3	6	0.35
	*Crocodylus niloticus*	6	1	12	0.61
	*Crocodylus novaeguineae*	1	n.a.	12	0.48
	*Crocodylus palustris*	3	1	17	0.64
	*Crocodylus porosus*	6	4	20	0.51
	*Crocodylus rhombifer*	4	4	2	0.59
	*Crocodylus moreletii*	2	1	4	0.64
Osteolaeminae	*Mecistops cataphractus*	3	n.a.	36	^b^0.34
	*Osteolaemus tetraspis*	6	1	5	0.71
Gavialinae	*Gavialis gangeticus*	6	5	19	0.28
Tomistominae	*Tomistoma schlegelii*	8	6	25	0.31
	total	110	55	256	

^a^*Caiman crocodilus crocodilus* + *C. c. fuscus*.

^b^Central African species [21].

**Figure 1.**
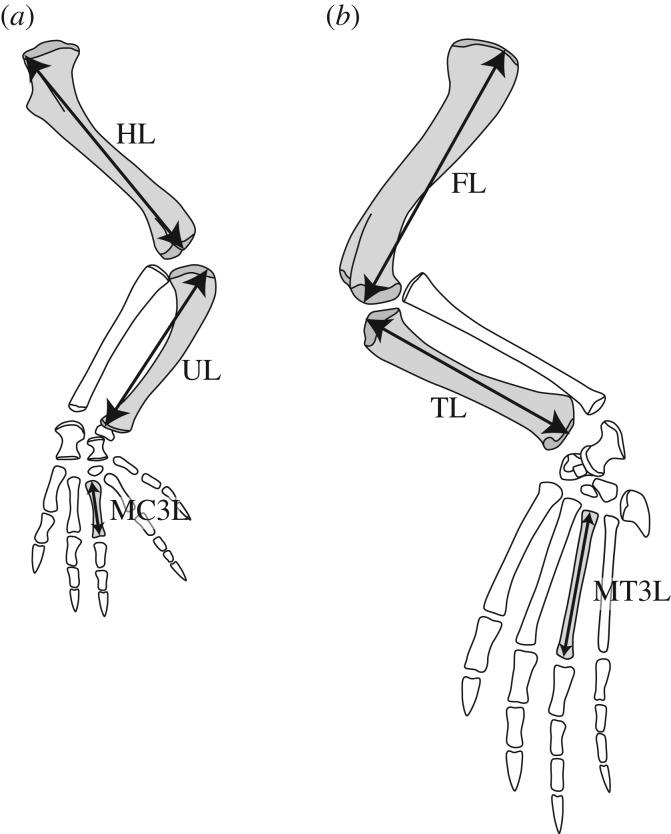
Measurements of forelimb (*a*) and hindlimb (*b*) long bones. FL, femur length; MC3 L, metacarpal III length; MT3 L, metatarsal III length; HL, humerus length; TL, tibia length; UL, ulna length.

In this study, maximum lengths of three main forelimb segments (humerus, ulna and metacarpal III: HL, UL and MC3 L) and three main hindlimb segments (femur, tibia and metatarsal III: FL, TL and MT3 L) were measured (figure 1). Measured bones were selected based on previous works on crocodylian limb proportions [24,25]. For the forelimb, the ulna instead of the radius was used because the ulna is much stouter than the radius and is assumed to be the major weight-bearing element among forelimb zeugopodium. The olecranon process of crocodylian ulna is minute and the degree of its development may have negligible effects on the total length. Measurements of fossil crocodyliforms were obtained by direct observation, through personal communications and from the literature (electronic supplementary material, table S3). Only either fore- or hindlimb was available for some individuals. When metacarpal III or metatarsal III length was not available, it was estimated from the average ratio of metacarpal II to III, or that of metatarsal II or IV to III of three extant taxa (*Melanosuchus niger*, *Caiman crocodilus* and *Crocodylus acutus* [22]). Lengths of left and right elements were averaged when possible.

We used the trunk length (sum of the 3rd cervical to the 15th dorsal centrum lengths) as a body length proxy, which was measured for 55 individuals of 17 species (table 1; electronic supplementary material, table S1) among the 20 extant species for which limb bones were measured. For fossil taxa, trunk lengths were measured only in *B. vorax, B. wilsoni*, and *N. mooki*.

Crocodylian rostral shape is known to be associated with their diets [17,26], feeding function and performance [27–29]. Here, rostral proportion, which is defined as the ratio of the palate width at the last maxillary alveoli to the length from there to the tip of the snout, was measured as a correlate of trophic function. The average rostral proportions were obtained for 20 species (table 1; electronic supplementary material, table S2). Small individuals with skull width less than 100 mm were excluded from the dataset. Fossil taxa were not included in the dataset because they are represented by a single or few individuals with various size classes, and their rostral shapes would be largely dependent on size.

### 2.2. Data analyses

As crocodylian limbs show ontogenetic allometry [24,25], size normalization with allometric correction of the measurements is required prior to species comparison. We used the geometric mean (GM) of six limb segments (GM = [HL × UL × MC3 L × FL × TL × MT3 L]^1/6^) as the size index for each individual, because the GM would more accurately reflect the overall body size than the length of a single bone (e.g. humerus or femur). All the measurements were normalized using the equation of Elliott *et al*. [30], which was adopted by recent vertebrate workers (e.g. [31–33]): *M*_s_ = *M*_o_(*L*_s_/*L*_o_)^*b*^, where *M*_s_ is the standardized measurement, *M*_o_ is the original measurement, *L*_s_ is the overall mean GM of all individuals, *L*_o_ is the GM of each individual and *b* is the regression slope of log_10_*M*_o_ (response variable) on log_10_*L*_o_ (predictor variable) for all individuals. If fossil taxa preserved either only fore- or hindlimb, the normalization was separately done for fore- and hindlimbs, using geometric means of segment lengths of forelimb (HL, UL and MC3 L) or hindlimb (FL, TL and MT3 L) as size indexes.

All normalized measurements (cHL, cUL, cMC3 L, cFL, cTL and cMT3 L) were log10-transformed to remedy the right-skewed distribution [34]. Principal component analysis (PCA) was used to reduce the dimensionality of the dataset, and the limb morphospace was visualized by a PC score plot. Correlations between size and the normalized measurements were tested by ordinary least squares (OLS) regressions of log_10_GM versus first three PC axes. To examine the difference in the multivariate means between the two superfamilies (Alligatoroidea and Crocodyloidea), non-parametric multivariate analysis of variance (npMANOVA: Bray–Curtis distance, 9999 permutations) was conducted for the log10-transformed normalized measurements using the R package ‘vegan’ [35]. The analysis was run separately for smaller (GM < median GM) and larger (GM ≥ median GM) subsamples to examine the size effect on limb measurements. Because the phylogenetic placement of Gavialinae (*G. gangeticus*) differs between molecular (e.g. [1–3,36,37]) and morphological (e.g. [4,38–40]) trees, we performed the alternative analysis excluding *G. gangeticus* from the dataset. The npMANOVA was also conducted for the whole sample using the subfamily as a factor, followed by pairwise comparisons for all pairs of subfamilies (*p*-values adjusted by the Holm method).

For the species comparisons, the normalized measurements were averaged within each species. To help understand the difference in the limb proportion among 20 extant and 14 fossil species, ternary diagrams of within-forelimb and within-hindlimb segment proportions, and a univariate plot of forelimb/(fore + hindlimbs) were provided. Evolutionary histories of fore- and hindlimb proportions were reconstructed on a recent comprehensive molecular tree of Oaks [3] using the contMap() function of the R package ‘phytools’ [41]. Phylogenetic relationships of fossil taxa are based on literature [40,42–44], disregarding the inconsistent placement of *G. gangeticus* between the molecular and morphological trees. Ages of fossil taxa were assumed to be the midpoints of their stage ranges, and root age was set at 129.4 Ma (beginning of Barremian [45]), which is the first appearance datum for the basalmost taxon, *A. hooleyi* [46]. Divergence times of extant taxa were adopted from Oaks' [3] species tree (90 Ma upper limit on the root age), and that of fossil taxa were estimated by dividing the ‘shared’ branch lengths equally [47]. Here, *A. gracilis* was regarded as a close relative of *A. haupti*, and *T. machikanensis* was regarded as a sister taxon to *T. schlegelii*.

Relative lengths of total fore- and hindlimbs against body length were obtained from extant crocodylians for further analyses. Allometry in trunk length (sum of 3rd cervical to 15th dorsal centrum lengths), total forelimb length (HL + UL + MC3 L) and total hindlimb length (FL + TL + MT3 L) were corrected by the above-described normalization equation, using the GM of the three measurements (GM = [trunk length × total forelimb length × total hindlimb length]^1/3^) as a size index. Subsequently, species average relative forelimb length (total forelimb/trunk length) and hindlimb length (total hindlimb/trunk length) were compared among 17 species (table 1; electronic supplementary material, table S1). We then tested if relative proximal segment lengths within fore- and hindlimbs (cHL/total forelimb; cFL/total hindlimb) are associated with relative fore- and hindlimb lengths (total forelimb/trunk length; total hindlimb/trunk length), by phylogenetically generalized least squares (PGLS) regressions for 17 species.

**Figure 2.**
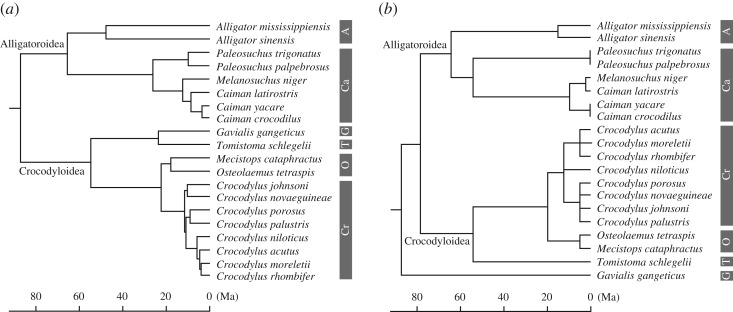
Time-calibrated molecular (*a*) and morphological (*b*) trees of Crocodylia used in this study. Subfamily abbreviations: A, Alligatorinae; Ca, Caimaninae; Cr, Crocodylinae; G, Gavialinae; O, Osteolaeminae; T, Tomistominae.

To explore the locomotor and trophic correlation in crocodylians, we performed PGLS regressions of rostral proportions (table 1; electronic supplementary material, table S2) versus relative proximal segment lengths (cHL/total forelimb; cFL/total hindlimb) using the average values of 20 extant species. Additionally, PGLS regressions of rostral proportions versus relative overall limb length ([fore + hindlimbs]/trunk length) for 17 extant species are performed. Because rostrum shape is size dependent (but not growth stage dependent) in extant crocodylians, and large forms attain an increasingly wider rostrum beyond a certain size (skull width approx. 240 mm for crocodyloids [48]), we have conducted PGLS regressions twice: either using all individuals with skull width ≥ 100 mm (*n* = 256), or using individuals within a set size range (100 mm ≤ skull width ≤ 200 mm; *n* = 151), to test the effect of size range on the results. In PGLS regressions, the scaling parameter lambda was estimated by the maximum likelihood method and incorporated into the regression, using the R package ‘caper’ [49]. The time-calibrated trees required for the PGLS regression were reconstructed in reference to a comprehensive molecular tree (figure 2*a*: [3]), with divergence times adopted from Oaks' [3] species tree (90 Ma upper limit on the root age). The alternative morphological tree (figure 2*b*) is based on Brochu [4,39,50], with divergence times estimated from first appearance data of fossils [4,51]. The root age (87.14 Myr) is adopted from Oaks [3]. In the morphological tree, relationships within Indo-Pacific *Crocodylus* are left unresolved, and *Mecistops* is regarded as a sister taxon to *Osteolaemus* based on the recent molecular and morphological evidence [2,3,21]. All statistical analyses were carried out with R language and environment [52]. Drawings of limbs and trunk (figures 1 and 4–7) are based on Mook [22] and Richardson *et al*. [53].

## 3. Results

**Figure 3.**
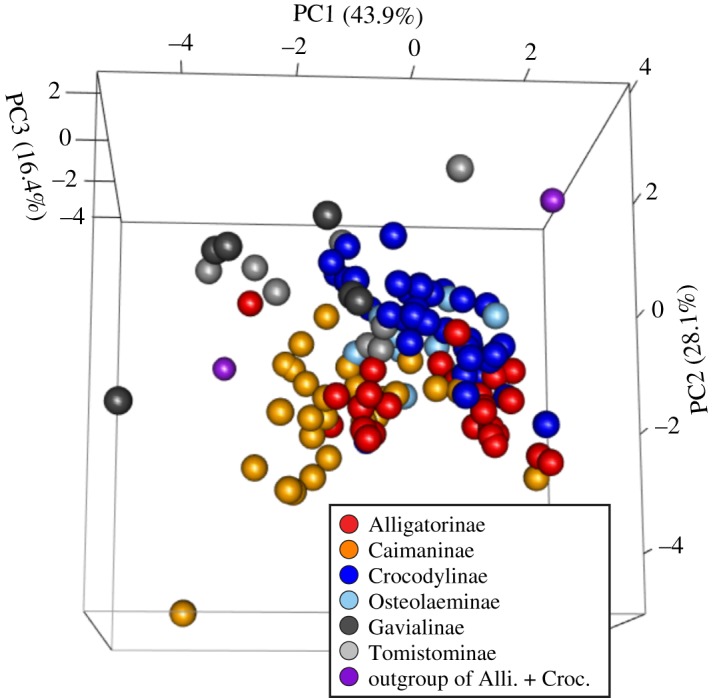
Three-dimensional component score plots generated from the PCA of log10-transformed normalized limb measurements. Alli., Alligatoroidea; Croc., Crocodyloidea.

PCA of log10-transformed normalized limb measurements revealed that about 90% of the variance is explained by first three PC axes (table 2). OLS regressions of log_10_GM against the first three PC axes indicated non-significant relationships between size and limb measurements (*p* = 0.66, 0.31, 0.80 for first, second and third axis, respectively). The first PC axis accounts for relative fore- versus hindlimb length. Individuals with high PC1 scores have longer humerus, ulna and metacarpal III and shorter femur, tibia and metatarsal III. The second PC axis describes relative contribution of stylopodium, zeugopodium and metapodium within fore- and hindlimbs. Individuals placed high on this axis have longer stylopodia (humerus and femur) and shorter metapodia (metacarpal III and metatarsal III). A three-dimensional PC score plot (figure 3) shows that alligatoroids (Alligatorinae and Caimaninae) and crocodyloids (Crocodylinae, Osteolaeminae, Gavialinae and Tomistominae) are moderately separated on the first and second axes. npMANOVA of the log10-transformed normalized limb measurements with smaller (GM < median GM) and larger (GM ≥ median GM) subsamples found significant differences between Alligatoroidea and Crocodyloidea (*F* = 13.643, *p* < 0.001 for smaller subsample; *F* = 8.399, *p* < 0.001 for larger subsample). The alternative test excluding *G. gangeticus* from subsamples also indicated significant differences (*F* = 13.833, *p* < 0.001 for smaller subsample; *F* = 7.342, *p* < 0.001 for larger subsample). Subfamily npMANOVA yielded a significant result (*F* = 13.697, *p* < 0.001). Pairwise comparisons among the subfamilies show significant differences (*p* < 0.05) for all subfamily pairs, highlighting the separation of limb morphospace among six subfamilies (electronic supplementary material, table S5).

**Figure 4.**
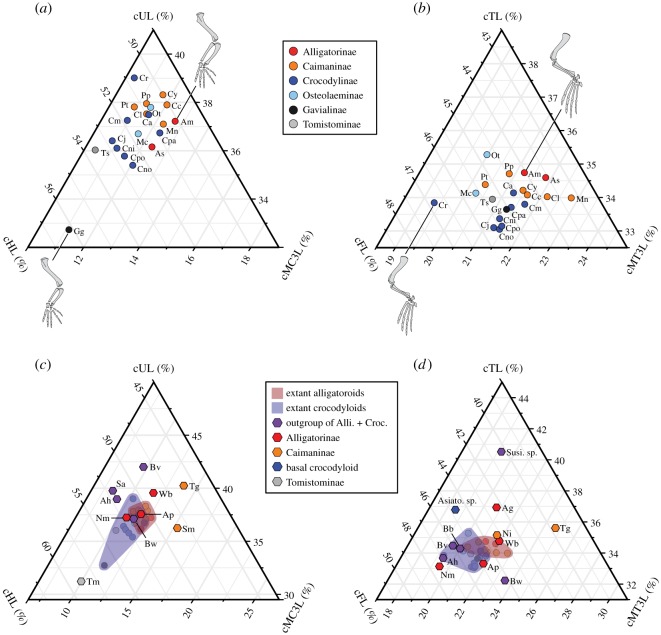
Comparisons of limb proportions among crocodylian species. Ternary diagrams of within-forelimb (*a*,*c*) and within-hindlimb (*b*,*d*) segment proportions for extant (*a*,*b*) and fossil (*c*,*d*) crocodylians. Alli., Alligatoroidea; Croc., Crocodyloidea. FL, femur length; MC3 L, metacarpal III length; MT3 L, metatarsal III length; HL, humerus length; TL, tibia length; UL, ulna length. Prefix ‘c’ (e.g. ‘cFL’) indicates normalized value. Species abbreviations: Ag, *Allognathosuchus gracilis*; Ah, *Anteophthalmosuchus hooleyi*; Am, *Alligator mississippiensis*; Ap, *Alligator prenasalis*; As, *Alligator sinensis*; Asiato, *Asiatosuchus*; Bb, *Boverisuchus bolcensis*; Bv, *Boverisuchus vorax*; Bw, *Borealosuchus wilsoni*; Ca, *Crocodylus acutus*; Cc, *Caiman crocodilus*; Cj, *Crocodylus johnsoni*; Cl, *Caiman latirostris*; Cm, *Crocodylus moreletii*; Cni, *Crocodylus niloticus*; Cno, *Crocodylus novaeguinea*e; Cpa, *Crocodylus palustris*; Cpo, *Crocodylus porosus*; Cr, *Crocodylus rhombifer*; Cy, *Caiman yacare*; Gg, *Gavialis gangeticus*; Mc, *Mecistops cataphractus*; Mn, *Melanosuchus niger*; Ni, *Necrosuchus ionensis*; Nm, *Navajosuchus mooki*; Ot, *Osteolaemus tetraspis*; Pp, *Paleosuchus palpebrosus*; Pt, *Paleosuchus trigonatus*; Sa, *Susisuchus anatoceps*; Susi, *Susisuchus*; Sm, *Stangerochampsa mccabei*; Tm, *Toyotamaphimeia machikanensis*; Tg, *Tsoabichi greenriverensis*; Ts, *Tomistoma schlegelii*; Wb, *Wannaganosuchus brachymanus*.

Ternary diagrams of fore- and hindlimbs in extant crocodylians demonstrate species’ differences in within-forelimb and within-hindlimb proportions (figure 4). As shown in the PCA for the whole sample, extant alligatoroids (Alligatorinae and Caimaninae) have a relatively short humerus within the forelimb elements (50.1–51.5% in Alligatorinae and 50.0–51.3% in Caimaninae), whereas extant crocodyloids without *Gavialis* (Crocodylinae, Osteolaeminae and Tomistominae) have a relatively long humerus (50.7–52.8% in Crocodylinae, 50.7–51.8% in Osteolaeminae and 53.6% in Tomistominae) (figure 4*a*). It is noteworthy that a gavialine, *G. gangeticus* exhibits by far the longest relative humerus within the forelimb elements (56.1%). The similar trend is observed in the hindlimb, where a relatively short femur within the hindlimb elements characterizes extant alligatoroids (43.6–44.0% in Alligatorinae and 43.2–45.2% in Caimaninae), while a relatively long femur is found in extant crocodyloids (44.5–46.8% in Crocodylinae, 44.7–45.6% in Osteolaeminae, 45.1% in Gavialinae and 45.3% in Tomistominae) (figure 4*b*).

**Table 2.** Factor loadings of log10-transformed normalized fore- and hindlimb measurements acquired from PCA for all individuals (*n* = 117).

log(variable)	PC1 (43.9%)	PC2 (28.1%)	PC3 (16.4%)
log(cHL)	0.250	0.800	0.492
log(cUL)	0.621	0.261	−0.692
log(cMC3 L)	0.708	−0.613	0.190
log(cFL)	−0.747	0.543	−0.027
log(cTL)	−0.810	−0.135	−0.403
log(cMT3 L)	−0.687	−0.535	0.254

Extinct species have explored much larger limb morphospace than the extant species. The ternary diagrams of limb segment proportions for fossils (figure 4*c*,*d*) reveal that most of the fossil alligatoroids (basal alligatorines *W. brachymanus* from the Palaeocene and *A. gracilis* from the Eocene and a derived alligatorine *A. prenasalis* from the Eocene), and three caimanines (*S. mccabei* from the Late Cretaceous, *N. ionensis* from the Palaeocene and *T. greenriverensis* from the Eocene) were plotted nearby extant alligatoroids in having relatively shorter stylopodia (cHL/total forelimb ≤ 50.8%; cFL/total hindlimb ≤ 44.9%), suggesting that the modern alligatoroid limb morphology was acquired in early times of their evolution (figures 4*c*,*d* and 6). An exception is a basal alligatorine, *N. mooki*, which has a relatively long humerus (52.1%) and femur (47.4%) similar to crocodyloids. The basal crocodyloid *Asiatosuchus* is plotted outside of the extant species range with a relatively long tibia. The tomistomine *T. machikanensis* is placed beyond the range of extant crocodyloids, in having extremely long humeri within forelimb elements (cHL/total forelimb = 58.8%). Outgroup taxa of Alligatoroidea + Crocodyloidea have diverse forelimb proportions (*B. vorax* with relatively short humerus and long ulna) and hindlimb proportions (*Susisuchus* sp. with short femur and long tibia, and *B. wilsoni* with short tibia and long metatarsal III).

**Figure 5.**
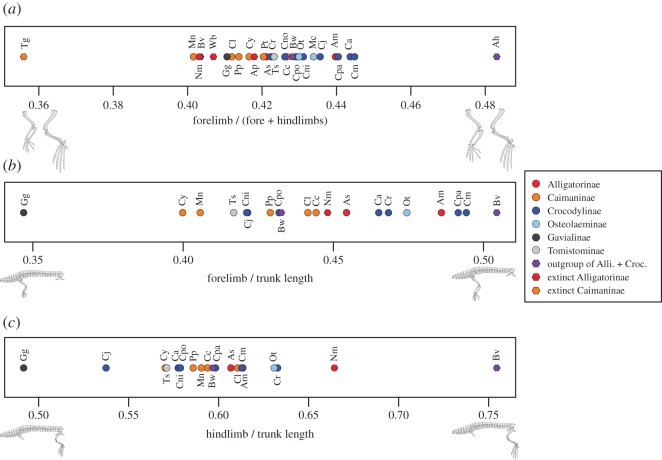
Univariate plots of forelimb/(fore + hindlimbs) (*a*), forelimb/trunk length (*b*), and hindlimb/trunk length (*c*). Alli., Alligatoroidea; Croc., Crocodyloidea. For species abbreviation, see legend of figure 4.

The fore- versus hindlimb proportion varies among and within subfamilies, where forelimb/(fore +hindlimbs) is relatively small in Caimaninae (35.6–42.7%) and Gavialinae (41.1%), relatively large in Crocodylinae (42.2–44.5%), Osteolaeminae (43.0–43.4%) and Tomistominae (42.3%), and largely varies around the average in Alligatorinae (40.3–44.0%) (figure 5*a*). The outgroup taxa are diverse in forelimb/(fore + hindlimbs), where *A. hooleyi* has an extremely long forelimb (48.3%). The comparison of relative fore- and hindlimb lengths to body length shows that *G. gangeticus* has the shortest fore- and hindlimbs relative to body length among all (figure 5*b*,*c*). Alligatoroids and crocodyloids have overlapping limb ratio ranges, but particularly in crocodyloids, it seems as though wider-snouted species (*Osteolaemus tetraspis*, *C. palustris*, *C. moreletii* and *C. rhombifer*) have longer limbs than the slender-snouted species (*T. schlegelii*, *C. johnsoni*, *C. acutus* and *C. porosus*; figure 7*e*).

**Figure 6.**
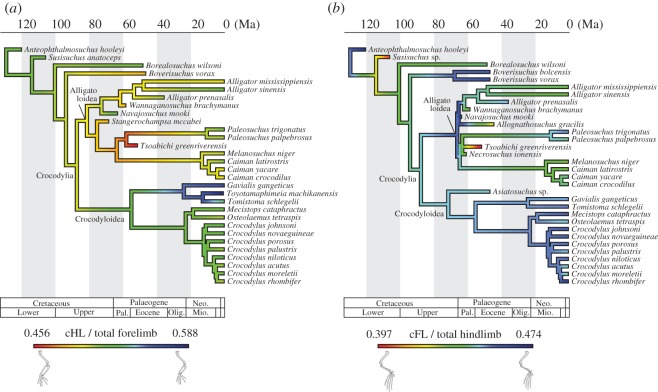
Evolution of limb proportions in crocodylians mapped on the phylogenetic tree. (*a*) cHL/total forelimb and (*b*) cFL/total hindlimb. FL, femur length; HL, humerus length. Prefix ‘c’ (e.g. ‘cFL’) indicates normalized value.

**Figure 7.**
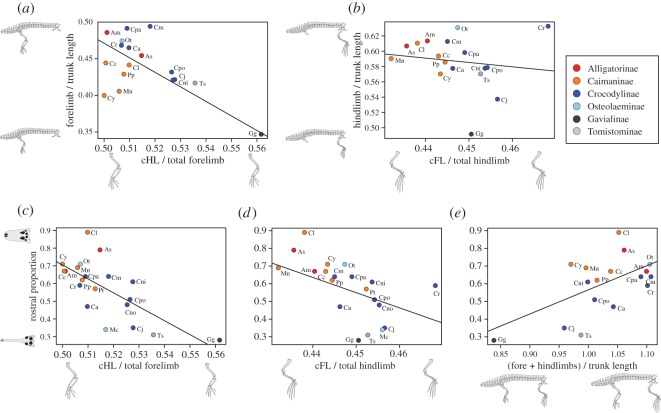
Results of PGLS regressions. (*a*) forelimb/trunk length against cHL/total forelimb. (*b*) hindlimb/trunk length against cFL/total hindlimb. (*c*–*e*) rostral proportion against cHL/total forelimb (*c*), cFL/total hindlimb (*d*), and (fore + hindlimbs)/trunk length (*e*). All PGLS regressions shown here used the molecular phylogeny (figure 2*a*: [3]). For species abbreviations, see legend of figure 4. FL, femur length; HL, humerus length. Prefix ‘c’ (e.g. ‘cFL’) indicates normalized value.

PGLS regressions of relative forelimb length (total forelimb/trunk length) on relative humerus length within forelimb (cHL/total forelimb) indicate that relative forelimb length to trunk length is significantly decreased in taxa with long humeri (adjusted *R^2^* = 0.498, *F* = 16.89, *p* = 0.001, *λ* = 0.520 for the molecular tree; adjusted *R^2^* = 0.451, *F* = 14.13, *p* = 0.002, *λ* = 0.5 for the morphological tree; figure 7*a*). The relationship between relative hindlimb length (total hindlimb/trunk length) and relative femur length within the hindlimb (cFL/total hindlimb) is non-significant (adjusted *R^2^* = −0.045, *F* = 0.316, *p* = 0.583, *λ* = 0 for the molecular tree; adjusted *R^2^* = −0.058, *F* = 0.13, *p* = 0.724, *λ* = 0.541 for the morphological tree) (figure 7*b*). Regressions of rostral proportions (individuals with skull width ≥ 100 mm) on limb ratio variables reveal significant negative correlations between rostral proportions and cHL/total forelimb (adjusted *R^2^* = 0.472, *F* = 17.95, *p* < 0.001, *λ* = 0 for both trees; figure 7*c*) and a weak correlation between rostral proportions and cFL/total hindlimb (adjusted *R^2^* = 0.151, *F* = 4.37, *p* = 0.051, *λ* = 0.449 for the molecular tree; adjusted *R^2^* = 0.336, *F* = 10.63, *p* = 0.004, *λ* = 0 for the morphological tree; figure 7*d*). Rostral proportions show significant positive correlation with (fore + hindlimbs)/trunk length (adjusted *R^2^* = 0.416, *F* = 12.4, *p* = 0.003, *λ* = 0.646 for the molecular tree; adjusted *R^2^* = 0.471, *F* = 15.25, *p* = 0.001, *λ* = 0.681 for the morphological tree; figure 7*e*). Alternative PGLS regressions using the average rostral proportion of each species with the upper size limit (100 ≤ skull width ≤ 200 mm) also show significant relationships between rostral proportions and limb ratio variables in the same manner (electronic supplementary material, table S6). These results indicate that slender-snouted taxa have relatively long stylopodia and short overall limbs (or long trunk).

## 4. Discussion

### 4.1. Difference of locomotor morphofunction between alligatoroids and crocodyloids

This study is the first to describe the interspecific variation in the limb proportion of crocodylians. Although the current sample shows a broad overall size range, the standardization procedure effectively removed size and ontogenetic allometry from the measurements, and major multivariate dimensions of limbs (PC1–3) did not show any correlation with size (log_10_GM). Therefore, if other sources of intraspecific variation, including sexual dimorphism [23,54] and phenotypic plasticity [16,55–58], are set aside, observed differences in the limb proportion would be largely attributed to their locomotor ecology and phylogenetic background.

Analyses of six fore- and hindlimb measurements demonstrated that crocodylian limbs are diverse, reflecting the phylogenetic history of each group. Two superfamilies as well as six subfamilies are separated in the multidimensional morphospace (figure 3; electronic supplementary material, table S5), contrary to the common wisdom that crocodylian postcranium is remarkably conservative [59,60]. Among the 20 species, *G. gangeticus*, a crocodyloid based on the molecular phylogenetic hypothesis, is placed close to *T. schlegelii* (Tomistominae) in the limb morphospace (figure 3), suggesting their similarity in locomotor ecology.

With regard to locomotor function, differences in the limb proportion between the two superfamilies (Alligatoroidea and Crocodyloidea) have an important implication. The segregation of the two superfamilies is seen in PC2 scores (figure 3) that reflect the relative contribution of stylopodia (humerus and femur) within fore- and hindlimbs: alligatoroids have relatively short stylopodia, whereas crocodyloids have relatively long stylopodia within each limb (figure 4). In addition, the relative lengthening of humerus within the forelimb is correlated with the overall forelimb length reduction (figure 7*a*).

The variation in the relative length of stylopodia and overall limb length observed in extant crocodylians is also seen through ontogeny of the American alligator, *Alligator mississippiensis*. Through growth, *A. mississippiensis* attains increasingly longer proximal limb segments relative to distal limb segments, with the decrease in relative overall limb lengths [24,25]. These ontogenetic changes are most probably associated with the reduced terrestrial locomotor capability in larger individuals of crocodylians [61–63]. Generally, lengthened proximal limb segments and shortened distal limb segments move muscle mass more distally along the limb. This would increase the moment of inertia of the limb about the shoulder and hip joint and reduce the velocity of limb protraction and retraction [24,64], although longer proximal limb segments may also indicate longer upper arm and thigh muscle fibres that would help quick limb motion [20]. Moreover, shortening of overall limb length compared to trunk length leads to a reduction of stride length [64]. Although limbs are used during slow paraxial swimming and bottom walking to some degree [65–70], they produce negligible propulsive force in fast axial swimming in crocodylians. Hence, short proximal limb segments and short overall limb length might be indicative of a reduced capability of terrestrial locomotion. In fact, *G. gangeticus*, arguably the most aquatic of living crocodylians [71–73], exhibits the longest humerus within forelimb and the shortest fore- and hindlimbs (figure 4). The difference in the limb proportion of alligatoroids and crocodyloids, specifically the relative contribution of stylopodia within fore- and hindlimbs, indicates that alligatoroids and crocodyloids may be different in their locomotor functions and habits. In accord with our findings, the morphology of the humerus–coracoid pair is clearly separated between the two living superfamilies: stouter humerus and coracoid and more abruptly emerging deltopectoral crest of alligatoroids are contrasted with the opposed character states in crocodyloids [18]. Fore- and hindlimb muscle properties are also different between the two groups, where extant crocodyloids generally have longer muscle fascicles especially in the pectoral limb and smaller muscle physiological cross-sectional areas than alligatoroids [20]. Because longer muscle fascicles can help achieve larger arcs of limb motion, this would be related to the use of asymmetrical gaits in crocodyloids [20]. However, smaller cross-sectional areas of crocodyloids' limb muscles may produce smaller forces, which would have an adverse effect on terrestrial locomotion.

The divergent locomotor morphofunctions seen in alligatoroids and crocodyloids might be deeply rooted in the crocodylian phylogeny (figure 6). Locomotor functions of the outgroup taxa of Alligatoroidea + Crocodyloidea, reflected in the proportions of their limb bones, were diverse and some species exhibit unique limb proportions that were not taken by extant crocodylians. Remarkably, *Susisuchus* and *B. vorax* show decoupled trends of fore- and hindlimb proportions, where extremely elongated zeugopodia (ulna or tibia) is observed for only either the fore- or hindlimb. For the ziphodont crocodylian *B. vorax*, an elongate ulna combined with another postcranial peculiarity (e.g. hoof-like ungual phalanges) [74] would suggest a terrestrial lifestyle of this taxon. After the split of Alligatoroidea and Crocodyloidea in the Late Cretaceous, most of the early alligatoroids from the Palaeocene and Eocene, such as *S. mccabei*, *A. gracilis*, *W. brachymanus*, *N. ionensis* and *T. greenriverensis* already acquired the limb proportions that are close to extant alligatoroids (relatively short humerus and femur). Only *N. mooki* from the Palaeocene is a notable exception to this trend in having a long humerus and femur similar to crocodyloids. Therefore, it is inferred that locomotor ecology of most of the early alligatoroids were similar to their living relatives. Crocodyloids, on the other hand, might explore the limb proportions towards longer stylopodia within fore- and hindlimbs, although their early evolution could not be confirmed with fossil taxa by the current sampling.

### 4.2. Locomotor and trophic integration

**Figure 8.**
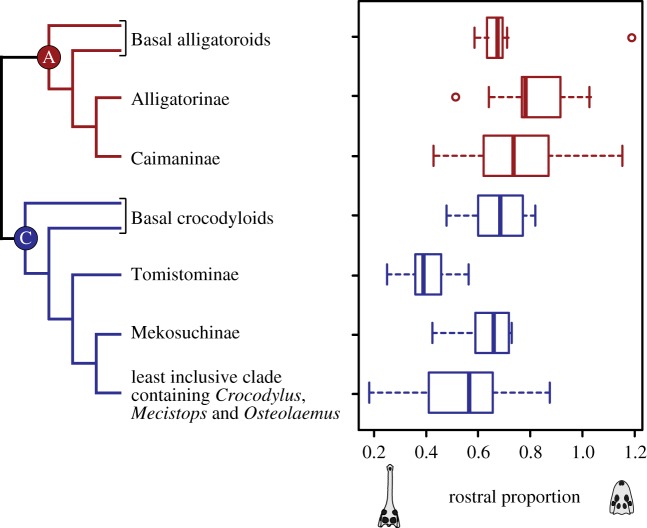
Distribution of rostral proportions among crocodylian subgroups on the phylogenetic tree (e.g. [44,75]). Enclosed A and C symbols refer to Alligatoroidea and Crocodyloidea, respectively.

The other ecological aspect worthwhile to discuss is the significant association of limb morphology and cranial shape. With larger proportions of stylopodia within fore- and hindlimbs (cHL/total forelimb; cFL/total hindlimb), and with shorter overall limbs relative to body length ([fore + hindlimbs]/trunk length), rostral shapes become narrower (figure 7*c–e*). Relatively long stylopodia and short overall limbs may indicate reduced terrestrial locomotor capability [24]. Because limbs do not play major roles in fast axial swimming, changes in limb proportions may negligibly affect aquatic locomotor capability in crocodylians [65–70]. On the other hand, the long and narrow rostrum enables higher angular velocity of the jaw tip during lateral head sweeping, and thus is favourable for catching small agile prey (i.e. fish) in water [27,28]. The correlation of appendicular and rostral proportions, which could be indicative of an association of locomotor and trophic functions, may have constrained morphofunctional diversification in crocodylians. To see if rostral shapes of crocodylians have diverged between alligatoroids and crocodyloids in similar fashion to that of the limb proportion, we here compiled the rostral proportions of 90 crocodylian species encompassing all the major clades of Alligatoroidea and Crocodyloidea (electronic supplementary material, table S4). Gavialoidea was not included in the dataset due to its contentious phylogenetic position (e.g. [1,4]). Because most fossil species are represented by few individuals, the size-related variation for each species was not accounted for here. The compilation shows that Tomistominae, Mekosuchinae and derived crocodyloids (i.e. the least inclusive clade containing *Crocodylus*, *Mecistops* and *Osteolaemus* in the morphological phylogenetic context) [75] have evolved slender-snouted forms beyond the range of alligatoroids (figure 8). On the contrary, basal alligatoroids, Alligatorinae and Caimaninae have taken the extreme broad rostral shapes that were never explored by crocodyloids. Of course, one should be cautious in interpreting the simple rostral proportion metric used here: apart from the slender–blunt evolutionary axis, crocodylians have other ways in transforming their skull shapes (ziphodont and duck-faced morphotypes [17]). Even so, the morphology of extant and fossil skulls implies that the trophic evolution of alligatoroids and crocodyloids is differently constrained in some way since their split in the Cretaceous, which is in accordance with the pattern of locomotor evolution illuminated by limb morphology (figure 6).

## 5. Conclusion

This study examined the morphofunctional difference in the fore- and hindlimbs of alligatoroids and crocodyloids. The difference in relative lengths of stylopodia indicated that alligatoroids and crocodyloids may be different in their locomotor functions, which is potentially related with terrestrial locomotor capability. Moreover, appendicular and rostral proportions are significantly correlated, where a longer forelimb stylopodium and short total limb lengths evolved in association with a slender snout, implying a possible connection between locomotor and trophic functions. This potential morphofunctional correlation might differently delimit the ecological opportunity of alligatoroids and crocodyloids, which seemingly have similar lifestyles in the present day water–land interface.

## Supplementary Material

Tables S1-S6

## Supplementary Material

Figure S1

## Supplementary Material

Supplementary references

## Data accessibility

The datasets supporting the conclusions of this article are included in the electronic supplementary material.
