# Supplementary material for: Comparative limb proportions reveal differential locomotor morphofunctions of alligatoroids and crocodyloids
Source: R Soc Open Sci. 2018 Mar 7;5(3):171774. doi: 10.1098/rsos.171774 (PMC5882705; doi:10.1098/rsos.171774)
Supplement: Supplementary references [file rsos171774supp3.docx]

**Electronic supplementary material**

**Supplementary references**

Aguilera OA, Riff D, Bocquentin-Villanueva J. 2006. A new giant *Purussaurus* (Crocodyliformes, Alligatoridae) from the Upper Miocene Urumaco Formation, Venezuela. Journal of Systematic Palaeontology 4: 221–232.

Antunes MT. 1961. *Tomistoma lusitanica*, crocodilien du Miocène du Portugal. Revista da Faculdade de Ciências Universidad de Lisboa, Serie II 9: 5–88.

Bartels WS. 1984. Osteology and systematic affinities of the horned alligator *Ceratosuchus* (Reptilia, Crocodilia). Journal of Paleontology 58: 1347–1353.

Berg DE. 1966. Die Krokodile, insbesondere *Asiatosuchus* und aff. *Sebecus*?, aus dem Eozän von Messel bei Darmstadt/Hessen. Abhandlungen des Hessischen Landesamtes für Bodenforschung 52: 1–105.

Bocquentin-Villanueva J, de Souza Filho JP, Buffetaut E, Negri FR. 1991. Nova interpretacao do genero *Purussaurus* (Crocodylia, Alligatoridae). Anais do XI Congresso Brasiliero de Paleontologia: 427–438.

Bonnan MF, Farlow JO, Masters SL. 2008. Using linear and geometric morphometrics to detect intraspecific variability and sexual dimorphism in femoral shape in *Alligator mississippiensis* and its implications for sexing fossil archosaurs. Journal of Vertebrate Paleontology 28: 422–431.

Brochu CA. 2004. A new Late Cretaceous gavialoid crocodylian from Eastern North America and the phylogenetic relationships of thoracosaurs. Journal of Vertebrate Paleontology 24: 610–633.

Brochu CA. 2007. Systematics and taxonomy of Eocene tomistomine crocodylians from Britain and northern Europe. Palaeontology 50: 917–928.

Brochu CA. 2010. A new alligatorid from the Lower Eocene Green River Formation of Wyoming and the origin of caimans. Journal of Vertebrate Paleontology 30: 1109–1126.

Brochu CA. 2011. Phylogenetic relationships of *Necrosuchus ionensis* Simpson, 1937 and the early history of caimanines. Zoological Journal of the Linnean Society 163: S228–S256.

Brochu CA. 2013. Phylogenetic relationships of Palaeogene ziphodont eusuchians and the status of *Pristichampsus* Gervais, 1853. Earth and Environmental Science Transactions of the Royal Society of Edinburgh 103: 521–550.

Brochu CA, Rincón AD. 2004. A gavialoid crocodylian from the Lower Miocene of Venezuela. Special Papers in Palaeontology, Fossils of the Miocene Castillo Formation, Venezuela: Contributions in Neotropical Palaeontology 71: 61–79.

Brochu CA, Storrs GW. 2012. A giant crocodile from the Plio-Pleistocene of Kenya, the phylogenetic relationships of Neogene African crocodylines, and the antiquity of Crocodylus in Africa. Journal of Vertebrate Paleontology 32: 587–602.

Buscalioni ÁD, Sanz JL, Casanovas ML. 1992. A new species of the eusuchian crocodile *Diplocynodon* from the Eocene of Spain. Neues Jahrbuch für Geologie und Paläontologie Abhandlungen 187: 1–29.

Cong L, Hou L, Wu X, Hou J. 1998. The gross anatomy of *Alligator sinensis* Fauvel. Beijing: Science Press.

Conrad JL, Jenkins K, Lehmann T, Manthi FK, Peppe DJ, Nightingale S, Cossette A, Dunsworth HM, Harcourt-Smith WEH, Mcnulty KP. 2013. New specimens of ’*Crocodylus*’ *pigotti* (Crocodylidae) from Rusinga Island, Kenya, and generic reallocation of the species. Journal of Vertebrate Paleontology 33: 629–646.

Delfino M, Smith T. 2009. A reassessment of the morphology and taxonomic status of ‘*Crocodylus*’ *depressifrons* Blainville, 1855 (Crocodylia, Crocodyloidea) based on the Early Eocene remains from Belgium. Zoological Journal of the Linnean Society 156: 140–167.

Delfino M, Piras P, Smith T. 2005. Anatomy and phylogeny of the gavialoid crocodylian *Eosuchus lerichei* from the Paleocene of Europe. Acta Palaeontologica Polonica 50: 565–580.

Densmore LD. 1983. Biochemical and immunological systematics of the order Crocodilia. In: Hechet MK, Wallace B, Prance GH, eds. Evolutionary Biology. New York: Plenum, 397–465.

Elliott NG, Haskard K, Koslow JA. 1995. Morphometric analysis of orange roughy (*Hoplostethus atalanticus*) off the continental slope of southern Australia. Journal of Fish Biology 46: 202–220.

Erickson BR. 1972. *Albertochampsa langstoni*, gen. et sp. nov., a new alligator from the Cretaceous of Alberta. The Scientific Publications of the Science Museum of Minnesota, New Series 2: 1–13.

Erickson BR. 1982. *Wannaganosuchus*, a new alligator from the Paleocene of North America. Journal of Paleontology 56: 492–506.

Erickson BR, Sawyer GT. 1996. The estuarine crocodile *Gavialosuchus carolinensis* n. sp. (Crocodylia: Eusuchia) from the late Oligocene of South Carolina, North America. The Science Museum of Minnesota, Monograph (Paleontology) 3: 1–47.

Erickson GM, Gignac PM, Steppan SJ, Lappin AK, Vliet, KA, Brueggen, JD, Inouye, BD, Kledzik, D, Webb, GJW. 2012. Insights into the ecology and evolutionary success of crocodilians revealed through bite-force and tooth-pressure experimentation. PLoS ONE 7: e31781.

Fabiani R. 1912. Contributi alla conoscenza dei vertebrati terziari e quaternari del Veneto: I. Il tipo del *Crocodilus vicetinus* Lioy. Memorie dell’Istituto geologico della R. Università di Padova 1.

Figueiredo RG, Kellner AWA. 2009. A new crocodylomorph specimen from the Araripe Basin (Crato Member, Santana Formation), northeastern Brazil. Palaontologische Zeitschrift 83: 323–331.

Figueiredo RG, Moreira JKR, Saraiva AÁF, Kellner AWA. 2011. Description of a new specimen of *Susisuchus anatoceps* (Crocodylomorpha: Mesoeucrocodylia) from the Crato Formation (Santana Group) with comments on Neosuchia. Zoological Journal of the Linnean Society 163: S273-288.

Fourtau R. 1920. Un nouveau genre de gavialidés. In: Department ES, ed. Contribution à l’étude des vertébrés Miocènes de l’Égypte. Cairo: Government Press, 116–121.

Gilmore CW. 1946. A new crocodilian from the Eocene of Utah. Journal of Paleontology 20: 62–67.

Hua S, Jouve S. 2004. A primitive marine gavialoid from the Paleocene of Morocco. Journal of Vertebrate Paleontology 24: 341–350.

Iijima M. 2017. Assessment of trophic ecomorphology in non-alligatoroid crocodylians and its adaptive and taxonomic implications. Journal of Anatomy 231: 192-211.

Jouve S, Bardet N, Jalil NE, Suberbiola XP, Bouya B, Amaghzaz M. 2008. The oldest African crocodylian: phylogeny, paleobiogeography, and differential survivorship of marine reptiles through the Cretaceous-Tertiary boundary. Journal of Vertebrate Paleontology 28: 409–421.

Jouve S, Bouya B, Amaghzaz M, Meslouh S. 2015. *Maroccosuchus zennaroi* (Crocodylia: Tomistominae) from the Eocene of Morocco: phylogenetic and palaeobiogeographical implications of the basalmost tomistomine. Journal of Systematic Palaeontology 13: 421–445.

Kobayashi Y, Tomida Y, Kamei T, Eguchi T. 2006. Anatomy of a Japanese tomistomine crocodylian, *Toyotamaphimeia machikanensis* (Kamei et Matsumoto, 1965), from the Middle Pleistocene of Osaka Prefecture: The reassessment of its phylogenetic status within Crocodylia. *National Science Museum Monographs* 35: 1–121.

Kraus R. 1998. The cranium of *Piscogavialis jugaliperforatus* n.gen., n.sp. (Gavialidae, Crocodylia) from the Miocene of Peru. Paläontologische Zeitschrift 72: 389–406.

Langston WJ. 1965. Fossil crocodilians from Colombia and the Cenozoic history of the Crocodylia in South America. University of California Publications in Geological Sciences 52: 1–152.

Langston WJ, Gasparini Z. 1997. Crocodilians, *Gryposuchus*, and the South American gavials. In: Kay RF, Madden RH, Cifelli RL, Flynn JJ, eds. Vertebrate Paleontology in the Neotropics: The Miocene Fauna of La Venta, Colombia. Washington D. C.: Smithsonian Institution, 113–154.

Lucas SG, Estep JW. 2000. Osteology of *Allognathosuchus mooki* Simpson, a Paleocene crocodilian from the San Juan Basin, New Mexico, and the monophyly of *Allognathosuchus*. New Mexico Museum of Natural History and Science Bulletin 16: 155–168.

Maccagno AM. 1948. Descrizione di una nuova specie di ‘*Crocodilus*’ del giacimiento di Sahabi (Sirtica). Atti della Reale Accademia Nazionale dei Lincei: Memorie della Classe di Scienze Fisiche, Serie 8 1: 61–96.

Martin JE. 2007. New material of the Late Cretaceous globidontan *Acynodon iberoccitanus* (Crocodylia) from southern France. Journal of Vertebrate Paleontology 27: 362–372.

Martin JE, Delfino M, Smith T. 2016. Osteology and affinities of Dollo’s goniopholidid (Mesoeucrocodylia) from the Early Cretaceous of Bernissart, Belgium. Journal of Vertebrate Paleontology: e1222534.

Medina CJ. 1976. Crocodilians from the late Tertiary of northwestern Venezuela: *Melanosuchus fisheri* sp. nov. Breviora 438: 1–14.

Mook CC. 1921a. Description of a skull of a Bridger crocodilian. Bulletin American Museum of Natural History 8: 111–116.

Mook CC. 1921b. Notes on the postcranial skeleton in the Crocodilia. *Bulletin of the American Museum of Natural History* 44: 67–100.

Mook CC. 1921c. The skull of *Crocodilus acer* Cope. Bulletin American Museum of Natural History 9: 117–121.

Mook CC. 1923. A new species of alligator from the Snake Creek Beds. American Museum Novitates 73: 1–13.

Mook CC. 1924. A new crocodilian from the Wasatch beds. American Museum Novitates 137: 1–4.

Mook CC. 1932. A study of the osteology of A study of the osteology of *Alligator prenasalis* (Loomis). Bulletin of the Museum of Comparative Zoology 74: 19–41.

Pickford M. 1994. Late Cenozoic crocodiles (Reptilia: Crocodylidae) from the Western Rift, Uganda. Geology and Palaeobiology of the Albertine Rift Valley, Uganda-Zaire, Voll. II–Palaeobiology. Orleans: CIFEG Occas Publication, 137–155.

Pickford M. 2003. A new species of crocodile from Early and Middle Miocene deposits of the lower Orange River Valley, Namibia, and the origins of the Nile crocodile (*Crocodylus niloticus*). Memoir of the Geological Survey of Namibia 19: 51–65.

Piras P, Buscalioni AD. 2006. *Diplocynodon muelleri* comb. nov., an Oligocene diplocynodontine alligatoroid from Catalonia (Ebro Basin, Lleida Province, Spain). Journal of Vertebrate Paleontology 26: 608–620.

Piras P, Delfino M, Favero, Letizia D, Kotsakis T. 2007. Phylogenetic position of the crocodylian *Megadontosuchus arduini* and tomistomine palaeobiogeography. Acta Palaeontologica Polonica 52: 315–328.

Price LI. 1964. Sóbre o crânio de um grande crocodilideo extinto do alto rio Juruá, Estado do Acre. Anais da Academia Brasiliera de Ciencias 36: 59–66.

Rauhe M, Rossmann T. 1995. News about fossil crocodiles from the Middle Eocene of Messel and Geiseltal, Germany. Hallesches Jahrbuch für Geowissenschaften 17: 81–92.

Revell LJ. 2012. phytools: An R package for phylogenetic comparative biology (and other things). Methods in Ecology and Evolution 3: 217–223.

Riff D, Aguilera OA. 2008. The world’s largest gharials *Gryposuchus*: description of *G. croizati* n. sp (Crocodylia, Gavialidae) from the Upper Miocene Urumaco Formation, Venezuela. Paläontologische Zeitschrift 82: 178–195.

Rossmann T. 1998. Studien an känozoischen Krokodilen: 2. Taxonomische Revision der Familie Pristichampsidae Efimov (Crocodilia: Eusuchia). Neues Jahrbuch für Geologie und Palaontologie Abhandlungen 210: 85-128.

Rossmann T. 2000. Studien an känozoischen Krokodilen: 5. Biomechanische Untersuchung am postkranialen Skelett des paläogenen Krokodils *Pristichampsus rollintii* (Eusuchia: Pristichampsidae). Neues Jahrbuch für Geologie und Paläontologie Abhandlungen 217: 289–330.

Sacco F. 1896. I coccodrilli del Monte Bolca. Memorie della Reale Accademia delle Scienze de Torino, Ser. 2 45: 75–88.

Salas-Gismondi R, Flynn JJ, Baby P, Tejada-Lara J V, Claude J, Antoine PO. 2016. A new 13 million year old gavialoid crocodylian from proto-amazonian mega-wetlands reveals parallel evolutionary trends in skull shape linked to longirostry. PLoS ONE 11: e0152453.

Salas-Gismondi R, Flynn JJ, Baby P, Tejada-Lara V, Wesselingh FP, Antoine PO. 2015. A Miocene hyperdiverse crocodylian community reveals peculiar trophic dynamics in proto-Amazonian mega-wetlands. Proceedings of the Royal Society B 282: 20142490.

Salisbury SW, Frey E, Martill D. 2003. A new crocodilian from the Lower Cretaceous Crato Formation of north-eastern Brazil. Palaeontographica. Abteilung A, Palaozoologie-Stratigraphie 270: 3–47.

Salisbury SW, Willis PMA. 1996. A new crocodylian from the Early Eocene of southeastern Queensland and a preliminary investigation of the phylogenetic relationships of crocodyloids. Alcheringa 20: 179–226.

Scheyer TM, Delfino M. 2016. The late Miocene caimanine fauna (Crocodylia: Alligatoroidea) of the Urumaco Formation, Venezuela. Palaeontologia Electronica 19.3.48A: 1–57.

Shan H yin, Wu X chun, Cheng Y nien, Sato T. 2009. A new tomistomine (Crocodylia) from the Miocene of Taiwan. Canadian Journal of Earth Sciences 46: 529–555.

Shirley MH, Vliet KA, Carr AN, Austin JD. 2014. Rigorous approaches to species delimitation have significant implications for African crocodilian systematics and conservation. Proceedings of the Royal Society B 281: 20132483.

Sill WD. 1970. Nota preliminar sobre un nuevo gavial del Plioceno de Venezuela y una discusion de los gaviales sudamericanos. Ameghiniana 7: 151–159.

Sternberg CM. 1932. The skull of *Leidyosuchus canadensis*. The American Midland Naturalist 13: 157–169.

Storrs GW. 2003. Late Miocene–Early Pliocene crocodilian fauna of Lothagam, southwest Turkana Basin, Kenya. In: Leakey MG, Harris JM, eds. Lothagam: the dawn of humanity in Eastern Africa. New York: Columbia University Press, 137–159.

Tchernov E. 1986. Evolution of the crocodiles in East and North Africa. Paris: Editions du CNRS.

Troedsson GT. 1924. On crocodilian remains from the Danian of Sweden. Lunds Universitet Årsskrift. N. F. 20: 1–75.

Vaillant L. 1872. Étude zoologique sur les crocodiliens fossiles tertiaires de Saint-Gérand-le-Puy. Annales de la Société Géologique de France 3: 1–58.

White TE. 1942. A new alligator from the Miocene of Florida. Copeia: 3–7.

Willis PMA. 1997. New crocodilians from the Late Oligocene White Hunter Site, Riversleigh, northwestern Queensland. Memoirs of the Queensland Museum 41: 423–438.

Willis PMA, Molnar RE. 1991. A new Middle Tertiary crocodile from Lake Palankarinna, south Australia. Records of the South Australian Museum 25: 39–55.

Willis PMA, Molnar RE. 1997. A review of the Plio-Pleistocene crocodilian genus *Pallimnarchus*. Proceedings of the Linnean Society of New South Wales 117: 224–242.

Willis PMA, Molnar RE, Scanlon JD. 1993. An Early Eocene crocodilian from Murgon, southeastern Queensland. Kaupia 3: 27–33.

Willis PMA, Murray PF, Megirian D. 1990. *Baru darrowi* gen. et sp. nov., a large broad-snouted crocodyline (Eusuchia: Crocodylidae) from mid-Tertiary freshwater limestones in northern Australia. Memoirs of the Queensland Museum 29: 521–540.

Wu X chun, Brinkman DB, Russell AP. 1996. A new alligator from the Upper Cretaceous of Canada and the relationships of early eusuchians. Palaeontology 39: 351–375.

Zangerl R. 1944. *Brachyuranochampsa eversolei*, gen. et sp. nov., a new crocodilian from the Washakie Eocene of Wyoming. Annals of the Carnegie Museum 30: 77–84.
